# It Is Really Simple: Foods and Human Health, The Whole Story

**DOI:** 10.3390/nu12072102

**Published:** 2020-07-16

**Authors:** Henry J. Thompson

**Affiliations:** Cancer Prevention Laboratory, Colorado State University, Fort Collins, CO 80523, USA; henry.thompson@colostate.edu; Tel.: +1-970-491-7748; Fax: +1-970-491-3542

**Keywords:** Western diet, metabolic disease, microbiome, inflammation, additives, acellular nutrients, whole foods, food industry, dietary guidelines, ultra-processed foods

## Abstract

Evidence continues to emerge that the gut-associated microbiome plays a central role in mediating the effects of diet on human health. A recent review of this field published in *Nutrients, “The Western Diet-Microbiome-Host Interaction and Its Role in Metabolic Disease”,* advances several dimensions of the diet–gut microbiome interaction that have received limited investigation. The intent of this editorial is to focus attention on components of processed foods, the consumption of which may alter the gut-associated microbiome in a manner that accounts for impacts on the immunity–inflammation axis that underlie the pathogenesis of obesity associated metabolic diseases. While examination of the issues that the authors articulate will take time, they unveil a simple fact—eating whole foods is an achievable path to health now. In saving this important observation until the end of their review, Zinöcker and Lindseth missed an opportunity to promote an important research strategy: use whole foods as the positive control in discerning those aspects of food processing that are detrimental versus being without effect.

## 1. Introduction

In an age of pandemics, it is important to retain focus on one pandemic that arguably is within our grasp to “cure”, i.e., the obesity pandemic. Obesity and the other chronic diseases to which it predisposes (e.g., type 2 diabetes, cardiovascular diseases, and a growing list of cancers) account for approximately 60% of deaths across the globe per annum, with profound personal and societal consequences and significant economic impacts in terms of health care costs and lost productivity [1,2]. For years, the causes of the obesity pandemic have been intensively investigated and many of the likely culprits have so frequently been discussed that the terms used to describe them have lost clarity [3,4]. Thus, it was “refreshing” to reflect on a recent review of this field published in *Nutrients, “The Western Diet-Microbiome-Host Interaction and Its Role in Metabolic Disease”* [5]. Zinöcker and Lindseth dissect the term the “Western diet” in a manner that can ignite much needed debate about the definition and role of processed foods in food patterns given the label “Western”. Perhaps more importantly, the ideas presented in their review can stimulate rapid re-analyses of existing data resources that contain food intake patterns and data on the gut-associated microbiome, as well as result in new studies of whole versus processed foods and their impact on host systemic processes such as inflammation as mediated by changes in the structure and/or function of the gut-associated microbiome.

Zinöcker and Lindseth make a compelling case for the importance of the role of diet on the development of metabolic disease through the lens of mediation by the gut-associated microbiome. However, I judge that the authors “underplayed” a key observation: the path to ending the obesity pandemic is simple: eat whole foods. This premise is in fact supported by a recent position paper of the American Heart Association [6]. In this editorial, I briefly restate key points made by Zinöcker and Lindseth in order to create a framework for discussion and to stimulate the research process.

## 2. Ultra-Processed Foods

Several sections of their review [5] addressed various aspects of the manner in which foods are “manufactured” relative to their capacity to alter the gut microbiome in a manner that directly affects metabolic diseases through alterations in the immunity–inflammation axis. The central argument is that ultra-processed foods, defined as “industrial formulations made entirely or mostly from substances extracted from foods, derived from food constituents, or synthesized in laboratories from food substrates or other organic source”, are the actual drivers of the Western Dietary pattern’s effect on obesity. The authors note that this position is not universally accepted, and in fact, many population studies do not explicitly consider this definition. However, what is attractive about their definition is the clarity that it brings to the topic. Three opportunities emerge from Zinöcker and Lindseth’s thesis: (1) argue about the definition of ultra-processed food to increase understanding in order to move the field forward; (2) define a specific list of ultra-processed foods from the most commonly used food frequency questionnaires and go back to existing databases, particularly those which also contain data on the microbiome and/or plasma metabolome and establish whether there are grounds for the ultra-processed foods hypothesis as stated in [5]; and (3) initiate new work, if warranted, with food frequency questionnaires specifically validated for measuring consumption of ultra-processed foods using a consensus definition of that term.

## 3. Other Food Processing Considerations

Zinöcker and Lindseth make a case for impacts of food processing, some of which occur in foods that will be not be categorized as ultra-processed. Succinctly these food processing alterations fall into four categories: (1) consumption of acellular nutrients, (2) effects of additives (emulsifiers and non-caloric artificial sweeteners, (3) microbial products formed during food handling and processing before a food is consumed, and (4) novel chemicals formed in a food during processing, e.g., advanced glycation end products. As the authors note, for a number of these factors, the supporting evidence is “thin” and much has been derived from pre-clinical experiments, making it important to establish translational relevance. Nonetheless, their synthesis of a complex literature provides clarity and a valuable framework for discussion, re-evaluation of existing data, and new investigations. I judge that it also serves two additional purposes: (1) provides insight into why whole food consumption is a key to food-associated health benefits, and (2) serves as a guide for consumer behaviors when they prepare whole foods for consumption in their kitchens.

## 4. Mediation

Zinöcker and Lindseth make a bold assertation that consumption of processed foods, particularly ultra-processed foods, drives obesity and obesity associated metabolic diseases via inflammation mediated by the interaction between consumed foods and the gut-associated microbiome. I underscore their point that investigation of the gut microbiome must go beyond the “whose home” analyses typified by assessment of the alpha and beta diversity of gut microbial ecology to the more important but less investigated question of “what are they doing” using metagenomics and metaproteomics [7]. Despite rapid progress in this area, it is important to highlight that 1) there is currently no consensus on what constitutes the healthy human gut microbiome [8], and 2) that rodent models of the gut microbiome from which many of the observations supporting the authors’ central thesis were made, while useful models, also have distinct limitations, as reported in [9]. Clearly, this review focuses attention an important and emerging area of scientific inquiry, but the thesis advanced by Zinöcker and Lindseth is far from being considered established.

## 5. Final Comment

It is widely recognized that the investigation of the diet–gut microbiome axis is bringing game-changing insights to understanding the role of food patterns in human health promotion and disease prevention. A recent review in *Nutrients, “The Western Diet-Microbiome-Host Interaction and Its Role in Metabolic Disease”,* advances several dimensions of the diet–gut microbiome interaction that have received limited attention. The authors not only highlight the importance of reconsidering the role of diet on the development of metabolic disease through the lens of mediation by the gut-associated microbiome, but also provide key elements of a framework for considering the role of foods and the manner in which they are “manufactured” in altering the gut microbiome in a manner that directly affects metabolic diseases through alteration in the microbiome–immunity–inflammation axis. Despite this landmark achievement, the authors understated a critical observation, which should be the mantra of all health professionals interested in promoting human health, “eat whole foods”.
